# Editorial: Advances in perinatal stem cells research and applications

**DOI:** 10.3389/fcell.2025.1725523

**Published:** 2025-11-07

**Authors:** Hoda Elkhenany, Qiang Wu, Ahmed Lotfy

**Affiliations:** 1 Department of Surgery, Faculty of Veterinary Medicine, Alexandria University, Alexandria, Egypt; 2 The State Key Laboratory of Mechanism and Quality of Chinese Medicine, Macau University of Science and Technology, Taipa, Macao SAR, China; 3 Department of Biology, Hal Marcus College of Science and Engineering, University of West Florida, Pensacola, FL, United States

**Keywords:** perinatal stem cells, amniotic mesenchymal stem cells, Wharton’s jelly, secretome, regenerative medicine, immunomodulation

**FIGURE 1:**
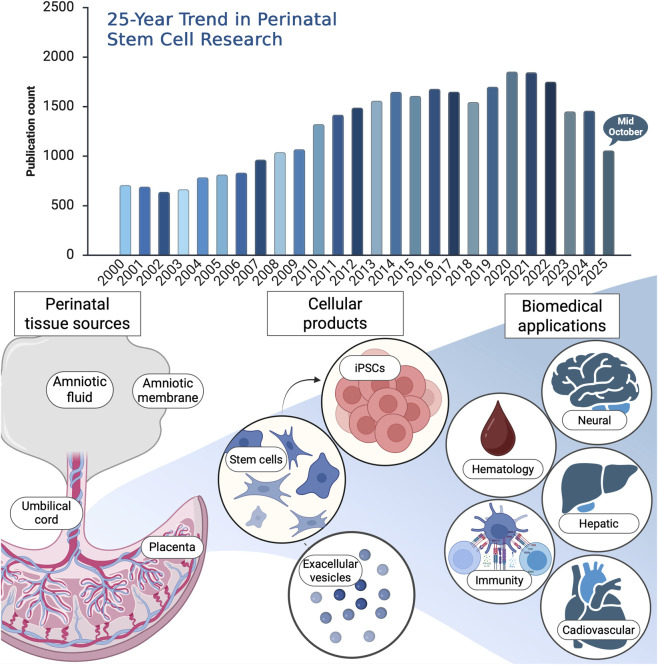
Upper panel: 25-year publication trend in perinatal stem cell research. Bibliometric analysis of publications on perinatal stem cells retrieved from the Web of Science Core Research Topic (search date: 15 October 2025). The search encompassed keywords covering perinatal stem cell types and related terms, including (“perinatal stem cell*” OR “perinatal mesenchymal stem cell*” OR “perinatal MSCs”) OR (“umbilical cord stem cell*” OR “umbilical cord blood stem cell*” OR “umbilical cord blood” OR “cord blood” OR “UCB”) OR (“Wharton’s jelly stem cell*” OR “Wharton’s jelly mesenchymal stem cell*” OR “WJ-MSCs”) OR (“amniotic stem cell*” OR “amniotic membrane stem cell*” OR “amniotic fluid stem cell*” OR “amniotic mesenchymal stem cell*” OR “AMSCs”) OR (“placental stem cell*” OR “placenta-derived stem cell*” OR “chorionic stem cell*”) OR (“perinatal tissue-derived cell*” OR “perinatal tissue-derived stem cell*”) OR (“perinatal secretome” OR “perinatal extracellular vesicles” OR “perinatal exosomes” OR “perinatal conditioned medium” OR “perinatal microRNA*” OR “perinatal miRNA”) OR (“fetal stem cell*” OR “fetal membrane stem cell*”). Lower panel: schematic overview of perinatal tissue stem cell sources and related cellular products, with emphasis on items published in this Research Topic. The diagram also highlights major biomedical application areas represented in the literature and in this issue top Research Topic include hematology and immunity, with additional coverage of neural, hepatic, and cardiovascular applications. Created with BioRender.

Over the past quarter-century, research on perinatal stem cells has expanded remarkably, reflecting their unique potential in regenerative and translational medicine. To capture the evolution of this field, a bibliometric analysis was conducted using the Web of Science (WOS) Core Research Topic (search date: 15 October 2025). The search strategy included major perinatal sources including umbilical cord, Wharton’s jelly, amniotic membrane, amniotic fluid, placenta, and fetal tissues as well as related secretome and extracellular vesicle studies. This comprehensive query yielded 55,639 publications, of which 40,131 were original research articles and proceedings papers. Notably, 33,273 publications appeared between 2000 and 2025, underscoring a steady and sustained rise in global scientific interest (Figure 1). The dominant research categories were Hematology and Immunology, followed by Cell Biology, Experimental Medicine, Tissue Engineering, and Oncology. These findings highlight not only the rapid expansion of perinatal stem cell research but also its broad interdisciplinary integration of fundamental biology to clinical translation and emerging biotechnologies.

Here in this Research Topic, the published articles closely mirror the global research trends revealed by the WOS analysis. Collectively, they explore both the cellular and molecular dimensions of perinatal stem cell biology, as well as their therapeutic potential across multiple organ systems. In the following sections, we briefly summarize the main findings of these publications.

Distinct miRNA profiles in human amniotic tissue and its vesicular and non-vesicular secretome (Chaves-Solano et al.). This study provides a comprehensive profiling of microRNAs (miRNAs) in human amniotic membrane (hAM) tissue and its secretome, distinguishing between vesicular and non-vesicular fractions across the reflected and placental regions. Using next-generation sequencing, the authors revealed distinct miRNA clusters linked to muscle proliferation, connective tissue development, and glial cell growth. These findings highlight the amnion as an active source of bioactive miRNAs and open new perspectives for understanding its role in tissue regeneration and perinatal communication.

Immune composition of the mononuclear cell fraction of human umbilical cord blood (Kikuta et al.). Offering the most detailed immune profiling to date, this study mapped the CD34^−^ mononuclear cell fraction of human umbilical cord blood (UCB) and compared it to adult peripheral blood using flow cytometry and single-cell RNA sequencing. The results revealed a predominance of naïve immune subsets, CD4^+^ and CD8^+^ T cells, recent thymic emigrants, and naïve B cells, indicating the immunological immaturity and tolerance of UCB. These findings underscore UCB’s unique value as an ethically accessible source of immune and stem-like cells for next-generation regenerative and immunomodulatory therapies.

Induced neural stem cells ameliorate blood–brain barrier injury in cerebral ischemia-reperfusion rats (Liang et al.). Expanding the translational potential of perinatal-derived cells, this study demonstrated that human placental mesenchymal stem cells can be reprogrammed into induced neural stem cells (iNSCs) capable of repairing ischemic brain injury. Transplanted iNSCs improved neurological outcomes and preserved blood–brain barrier integrity by modulating astrocytic calcium signaling, reducing oxidative stress, and suppressing apoptosis. These results position iNSCs as a promising, ethically favorable source for neurovascular regeneration in ischemic stroke.

Amniotic mesenchymal stem cells attenuate diabetic cardiomyopathy by inhibiting pyroptosis via modulation of the TLR4/NF-κB/NLRP3 pathway (Zhou et al.). This study highlights the cardioprotective potential of amniotic mesenchymal stem cells (AMSCs) in diabetic cardiomyopathy (DCM), a major complication of diabetes with limited therapeutic options. Using a diabetic mouse model, repeated AMSC administration improved glucose tolerance, insulin secretion, and overall cardiac performance. Mechanistically, AMSCs suppressed pyroptosis and inflammation through inhibition of the TLR4/NF-κB/NLRP3 signaling cascade and attenuated myocardial fibrosis by modulating the TGF-β/Smad pathway. These findings emphasize the dual metabolic and anti-inflammatory actions of AMSCs and position them as a promising regenerative therapy for DCM, warranting further investigation into long-term efficacy and mechanisms of action.

Wharton’s jelly mesenchymal stromal cells derived from preterm umbilical cord reveal a hepatogenic potential (Timoneri et al.). This study provides novel insights into the developmental influence on the regenerative capacity of Wharton’s jelly mesenchymal stromal cells (WJ-MSCs). By comparing preterm and term umbilical cord–derived WJ-MSCs, the authors demonstrated that preterm cells possess a markedly higher hepatogenic potential, differentiating more efficiently into hepatocyte-like cells (HLCs) with enhanced expression of hepatic markers and superior functional maturity. Transcriptomic profiling further revealed an enrichment of pluripotency-associated genes and signaling pathways favoring hepatic lineage specification in preterm WJ-MSCs. These findings identify the preterm umbilical cord as a particularly promising source for liver regeneration, offering a developmentally primed cell population for future hepatocellular therapies.

In conclusion, the articles in this Research Topic underscore the growing maturity and translational promise of perinatal stem cell research. From exploring molecular regulators such as miRNAs to advancing preclinical applications in neural, cardiac, and hepatic regeneration, these studies exemplify the multidimensional potential of perinatal tissues as accessible, ethically sound, and biologically versatile sources for next-generation therapies. Moving forward, the integration of omics-driven profiling, standardized cell products, and mechanistic *in vivo* validation will be essential to bridge laboratory discoveries with clinical translation.

